# Reports on the Hospitals of the United Kingdom

**Published:** 1910-04-02

**Authors:** Henry Burdett


### REPORTS
ON THE
### Hospitals of the United Kingdom.

By SIR HENRY BURDETT, K.C.B., K.C.V.O.

### XXXV.—LEATHERHEAD (VICTORIA MEMORIAL) COTTAGE HOSPITAL.

This hospital contains eight beds in two wards of three beds each and a small ward. It was built as a memorial to Queen Victoria, and was opened about four years ago. We inspected it in September, and were pleased to find marked efficiency everywhere except in certain details in the architect's work. The elevation seems to have been the first consideration of the architect, for the w.c.s have windows in the wrong place and at the wrong level, and the linen-room and larder have no ventilation at all. The latter omissions, which we have found to be a prominent feature in architect work, can be easily remedied by putting a grating of sufficient size at the floor level in each room communicating with the outer air, and one in the ceiling opening into the open roof.

The great need of this hospital is a large verandah running the whole length of the building on the garden side, which should be at least 10 feet wide, with a concrete floor and an iron and glass roof. Here patients might sleep in the open air with great benefit to themselves and great addition to the popularity and usefulness of this hospital. Seeing the number of wealthy residents in the neighbourhood, there will surely be some among them who will grasp the privilege of providing such a verandah without delay.

There is no mortuary at present, but one is about to be built. We hope that it will be carefully planned.

Miss Andrews, the matron, and the Committee are to be congratulated upon the present excellent condition of their little hospital.

### XXXVI.—THE BRIDGEWATER HOSPITAL.

This hospital was established in 1813. It occupies an old but roomy house, and is fairly well adapted for its purpose. There is an idea to rebuild the whole hospital in 1913, but we would counsel the reconstruction of portions of the present buildings, so as to make them sufficient for the hospital requirements of the population it serves. The hospital contains 42 beds, and some of the wards are pleasant and airy. The wards, almost necessarily, are not well arranged, but they are reasonably well kept, and the smaller wards are good. It is desirable to refloor portions of the wards if the present building is continued as a hospital, which we feel it properly may be, providing from £1,000 to £2,000 is expended in reconstruction under skilled and competent advice.

The accommodation for the nurses is not good. Three rooms open one into the other, so that there can be neither adequate privacy nor comfort for the nurses who occupy these rooms. The nurses' bath, too, is small and inadequate, and better bathing arrangements for the resident staff, and also for the matron, ought to be provided at once.

An attempt is being made here to abolish the out-patient department, a step not popular with the poorer residents who use this hospital. We would counsel the appointment of a small active and influential special committee to consider all the points we have mentioned, with a view to the preparation of a complete scheme for a new and perfected hospital, either by reconstruction, as we have suggested, or by rebuilding. We favour the former plan as being less costly and quite adequate, providing the best expert advice be sought.

### XXXVII.—WELLS GENERAL HOSPITAL.

This hospital was established in 1874 and rebuilt in 1895. It contains twelve beds. We presume that it was not designed by an architect who had had experience in hospital building. The staircase, for instance—all the wards save one being upstairs—was so constructed as to make it practically impossible for patients to be carried to the wards until quite recently, when it was reconstructed. The office accommodation is ample, but the fittings are common and not up to date. The twelve beds, and one cot, are contained in two large wards of five beds each and three small wards. The management is entrusted to the matron, Miss M. E. Smith, who has been a Queen's nurse and is full of energy and resource. She has only one assistant, who is not a trained nurse, and in consequence the matron had been on duty two days and one night without sleep when we inspected the hospital on September 1, 1909. It has happened that the matron has been on duty, when the hospital was full, three nights and four days at a stretch. These things ought not to be, and when we find from the report that the subscriptions and general support extended to the hospital are very meagre, and that the income, such as it is, shows a tendency to decrease rather than to increase, we feel justified in saying that the committee should be aroused to greater energy by the independent action of the governors, who ought to take up the matter properly, and appoint a special committee of inquiry to take prompt action with the object of infusing more energy and vigour into the administration. As matters stand at present the impression we gained was that the cathedral authorities, from the Bishop downwards, ought to arouse themselves, if it is ever possible in the climate of Wells to be quite awake, and for the credit of the ancient foundation to put this little hospital on a secure basis.

The **Manchester Royal Infirmary** will be reported on next week.

